# Therapeutic potential of neural stem cells: greater in people's perception than in their brains?

**DOI:** 10.3389/fnins.2014.00079

**Published:** 2014-04-16

**Authors:** Elena Cattaneo, Luca Bonfanti

**Affiliations:** ^1^Department of BioSciences, Center for Stem Cell Research, University of MilanMilano, Italy; ^2^Department of Veterinary Sciences, University of TurinTorino, Italy; ^3^Neuroscience Institute Cavalieri Ottolenghi, University of TurinOrbassano, Italy

**Keywords:** brain repair, neurodegenerative diseases, regenerative medicine, science communication, science and society, press release, hope, therapeutic illusion

The discovery that the mammalian brain contains neural stem cells, and that such cells produce new neurons during adulthood through a process known as “adult neurogenesis,” led to the hypothesis that their underlying biology could be exploited for central nervous system (CNS) repair purposes. Yet, in spite of the large amount of knowledge gathered on adult neurogenesis during the last two decades, no substantial translational advances have been reached for most neurological diseases (Rossi and Cattaneo, 2002; Arenas, 2010; Lindvall and Kokaia, 2010). This may be linked to the intrinsic complexity of the nervous tissue at the structural, developmental, and evolutionary level, that preclude cell regeneration and repair (Weil et al., 2008; Bonfanti, 2011). As a result, neural cell replacement is at present not possible neither by implementation of endogenous neurogenic potential nor through transplantation of highly neurogenic stem cell sources.

However, in the gold rush-like phenomenon that has followed the discovery of neural stem cells the scientific community appeared often unaware of the limits associated with endogenous cell replacement and underestimated some problems and hurdles, which remain unknown to the general public and are sometimes undetected by the scientists themselves. In addition, the misleading communication of a series of basic scientific results that are often biased toward their possible translation, along with an amplification of interpretation from the media to the public have led to the perception that new therapeutic “possibilities” were at reach. What has been missed is a real vision of the state of the art: the present inability of medical science and fundamental research to overcome the gap between *in vitro* stem cell behavior and their adaptation to brain environment, as well as the need of more fundamental research prior to proposing therapeutic perspectives. As a consequence, the complex intermix emerging from stem cell discoveries, scientists' press releases, media, and society has produced substantial failure in the communication of scientific results, ultimately putting pressure toward premature translation of still insufficient pre-clinical data. In this Opinion article we suggest that a better communication from scientists to the public, without distortion by media and scientists themselves, is needed to avoid misunderstanding and to increase trust and constructive dialogue between science and society.

## Stem cell discoveries, adult neurogenesis, and the translational gap

Since the beginning of the nineties, the fact that adult neurogenesis can occur in mammals, at least within certain brain regions, led many scientists to exploit this endogenous capacity of the CNS for reparative/regenerative strategies. In parallel, several attempts have been put in place to bring the neural stem/progenitor cells in a dish or to obtain them from pluripotent stem cells. Yet, very small advances in the translation of this knowledge toward brain repair have been achieved. Many breakthroughs have been obtained in culture systems, thus increasing our ability to grow and manipulate stem cells (from the old neurosphere assay to the most striking example of the induced pluripotent stem cells—iPS—from the Yamanaka lab or of the direct reprogramming strategy). Yet, what hampers the possible therapeutic exploitation of stem/progenitor cells is a gap of knowledge between their activity/product and the host tissue environment. Paradoxically, the discovery of adult neurogenesis as a process which destroys the dogma of a static brain also shows that the exceptions are restricted to small neurogenic sites. Moreover, spontaneous neurogenesis in adult mammals is primarily linked to homeostatic roles and hardly directed to repair (Bonfanti, 2011). In other words, endogenous stem cells work well in their niches as do isolated stem cells *in vitro*, both sources of progenitors failing to properly act in the mature nervous tissue (normal and pathological). Hence, particularly in the CNS, unlike tissues that undergo continuous cell renewal (e.g., skin, blood, etc.), it is not granted that the goal of brain repair could be solved by gaining additional knowledge and/or by improving the availability of highly neurogenic stem cell sources.

Today, excellent protocols have been developed that recapitulate human brain development *in vitro* starting from human embryonic stem cells or human iPS cells. In the most extraordinary achievement, authentic dopaminergic neurons have, for the first time, been obtained which are capable of persistent functional recovery when transplanted into a mouse and rat model of Parkinson's disease (Kriks et al., 2011). In spite of such progress, it is still unclear how these cells which are highly plastic in culture systems may function and persist long-term *in vivo* and whether a permanent reconstruction of damaged brain circuits in adulthood may be realistic (Rossi and Cattaneo, 2002). Furthermore, quite surprisingly, in the large literature on adult neurogenesis and neural stem cells there are a few reports analyzing the factors hampering brain repair following the use of exogenously delivered and/or endogenous stem/progenitor cells; most studies are focused on the other side of the coin, e.g., the factors promoting neurogenesis.

While pursuing the cell replacement strategy, a further therapeutic approach might be that of exploiting the neuroprotective and immune modulatory capacities of both transplanted (Martino et al., 2011) and endogenous (Kokaia et al., 2012) stem/progenitor cells. Yet, the recent discovery of such “bystander effect” foreseeing transplanted cells as biological minipumps able to release beneficial factors, although promising under the profile of research, can be a further source of confusion between scientists, physicians, and patients. At the present time, more than 300 clinical trials have been started in the world to test the effect(s) of different stem cell sources in neurodegenerative diseases (Donegà et al., 2013). In most cases, little is known about the mechanisms by which different stem cell lines are expected to function *in vivo*, what is their survival and distribution, what are the beneficial (rather than detrimental) factors they release, whether their release is sustained over time or, rather, represent an acute reaction after transplantation which may vanish at prolonged survival times, and which aspect of the neuropathology is expected to be targeted.

In conclusion, it is our perception that the translation of basic knowledge discoveries on both endogenous and exogenous stem cells into robust, biologically, and therapeutically relevant cell-based strategies for neurological diseases still remains a far-reaching goal which requires further years of research, possibly with the notable exception of Parkinson's disease. In parallel, current adult neurogenesis research, although largely not tailored toward restoration and regeneration, might yield new perspectives through insight into fundamental principles of plasticity and particular roles of new neurons in the brain, most notably in the hippocampus (Kheirbek et al., 2012; Snyder and Cameron, 2012; Freund et al., 2013). Yet, when communicating science, a clear distinction should be made between translational perspectives having indirect implications for our understanding of aging and brain disease, and those directed at obtaining cell replacement goals.

## Stem cell discoveries: scientists, media, and society

As for many other fields, the stem cell arena has been characterized by repeated breakthroughs resulting in the public perception that “stem cells” act like magic bullets that can cure many diseases. Because of this high level of expectation, it has become increasingly difficult to maintain a balance between crude reality, realistic perspectives, and unjustified hopes. This failure in the dissemination of complex scientific concepts depends on the actions carried out by people bringing different kinds of responsibilities at various levels of the communication process. In particular, during the communication process, a number of significant steps may be overlooked as follows: (i) the original communication of the results obtained within the scientific community (scientific papers) is often too unbalanced toward the possible translation of basic science discoveries; (ii) there is an overemphasized release of information concerning the publication of a paper to the media; (iii) the above is followed by an amplification of interpretation/communication from the media to the public (which always stresses the “possible”—but at present non-existing—therapeutic use of a new discovery without paying any attention to the discovery process and to the value of the knowledge-acquisition process); (iv) patients and/or their families are often exposed to simplified interpretations of new therapeutic “possibilities”; (v) there is frequent misunderstanding about the real role of clinical trials in ascertaining the effectiveness and security of a given cell-based strategy; (vi) the recent description of the so called “bystander effect” has become a further source of overestimation of stem cell potential by incompetent physicians. What is not clearly perceived is the inability of medical science and fundamental research to overcome the gap between stem cell potential to regenerate new neurons and the non-permissive CNS environment preventing cell integration. Ultimately, a general thinking has prevailed supporting the view that stem cell treatments can produce new neurons in the diseased brain or that the bystander effects of given stem cells are already “therapies.”

A consequence of such misunderstanding has been particularly deleterious in Italy, where last year political decisions have led to the authorization by the Parliament of the first governmental sponsored clinical trial intended to evaluate an unverified cell-based “treatment.” This treatment was without a scientific basis, ineffective, and dangerous. Such a decision has been taken under the pressure of a small group of protesting lay people and patients without involving scientists and experts in the field. The State-sponsored trial has been stopped by the current Ministry of Health on the basis of a negative evaluation by an *ad hoc* appointed scientific committee. However, as we write, a regional court declared that committee unlawfully biased, and a new committee has now been nominated. Protesters and patients, however, are still in tribunals, several of them having authorized the injection of the same, unproven, unknown, and ill-prepared “stem cell potion” into people with diseases as severe as Parkinson, Amyotrophic Lateral Sclerosis, Spinal Muscular Atrophy, or even coma on the basis of a “constitutional right for a cure.”

## Conclusions and possible solutions

There are several gaps and distortions in the process of science communication on regenerative medicine in general—and for neurological diseases in particular—that can lead to serious abuses, misunderstanding, and heavy consequences for the patient's life. It is necessary to address such issues in the future by identifying the single steps and responsibilities in order to prevent/overcome the problem. One possible solution might be the introduction of an additional regulation of stem cell therapies, as suggested in a recent paper (Bianco et al., 2013): “The scientific community must consider the context—social, financial, medical, legal—in which stem cell science is currently situated and the need for stringent regulation. Additional concerns are emerging. These emanate from the novel climate, created within science itself, and stem cell science in particular, by the currently prevailing model of “translational medicine.” Only rigorous science and rigorous regulation can ensure translation of science into effective therapies rather than into ineffective market products, and mark, at the same time, the sharp distinction between the striving for new therapies and the deceit of patients.”

Yet, such a remedy might be ineffective in the absence of a more direct involvement of scientists into the communication process. In this context, the biological issues involved in the process of adult neurogenesis viewed as an *in vivo* product of neural stem cell activity, are even more tight to be grasped. The different ways by which spontaneous and/or lesion-induced neurogenic plasticity might be exploited for therapeutic strategies must be clearly explained to the public, as well as it should be made clear that long-term efforts are required to test how effective such strategies might be. Recently, the neuroscientist David M. Eagleman focused on the dilemma: “Communicating science to the public takes time away from busy research careers. So why would you do it?” (Eagleman, 2013). His answer consisted of six reasons which embrace the whole meaning of science and its beauty, from the need to acknowledge the funders, to stopping the flow of bad information, to the need of clarifying what science is and is not. These tasks should remain within the responsibilities of scientists themselves because that is exactly what they are “well set up for.”

### Conflict of interest statement

The authors declare that the research was conducted in the absence of any commercial or financial relationships that could be construed as a potential conflict of interest.
